# Fecal Microbiota Transplantation in Patients Co-Infected with SARS-CoV2 and *Clostridioides difficile*

**DOI:** 10.3390/biomedicines11010007

**Published:** 2022-12-21

**Authors:** Adrian Boicean, Bogdan Neamtu, Sabrina Birsan, Florina Batar, Ciprian Tanasescu, Horatiu Dura, Mihai Dan Roman, Adrian Hașegan, Dan Bratu, Alin Mihetiu, Călin Ilie Mohor, Cosmin Mohor, Ciprian Bacila, Mihai Octavian Negrea, Sorin Radu Fleaca

**Affiliations:** 1 County Clinical Emergency Hospital of Sibiu, 550245 Sibiu, Romania; 2 Faculty of Medicine, Lucian Blaga University of Sibiu, 550169 Sibiu, Romania; 3 Pediatric Research Department, Pediatric Clinical Hospital Sibiu, 550166 Sibiu, Romania; 4 Dr. Gheorghe Preda Psychiatric Hospital of Sibiu, 550082 Sibiu, Romania

**Keywords:** fecal microbiota transferring, co-infection, SARS-CoV2, *C. difficile*

## Abstract

Background: The COVID-19 pandemic has challenged the treatment of *Clostridioides Difficile* (CD)-infected patients given the increasing number of co-infections with *severe acute respiratory syndrome coronavirus* 2 (SARS-CoV-2). In this context, fecal microbiota transplantation (FMT) shows promise in modulating the immune system’s function and alleviating the burdens associated with this condition. Methods: To achieve this goal, we performed a comparative, retrospective, single-center study on 86 patients (admitted between January 2020 and March 2022). We based our approach on specific inclusion criteria: 1. The study group included 46 co-infected patients (COVID-19 and CD) receiving antibiotics and FMT; 2. In the control group, 40 co-infected patients received antibiotics only. Our results showed no significant group differences in terms of gender, age, risk factors such as cardiovascular and neurological diseases, type 2 diabetes, and obesity (*p* > 0.05), or in pre-treatment inflammatory status, evaluated by white blood cell (WBC) count and C-reactive protein (CRP) levels. We report a significant decrease in inflammatory syndrome (CRP, WBC) in coinfected patients receiving FMT in addition to antibiotics (*p* < 0.05), with a lower relapse rate and mitigation of cramping and abdominal pain (91.3%). In addition, a higher level of fibrinogen, persistent moderate abdominal pain (82.5%), and a significantly higher CD infection relapse rate (42.5%) were recorded in co-infected patients treated only with antibiotics (*p* < 0.05). Conclusion: Our study provides new data to support the multiple benefits of FMT in the case of COVID-19 and CD co-infection by improving patients’ quality of life and inflammatory syndrome.

## 1. Introduction

The COVID-19 pandemic has challenged the management of *severe acute respiratory syndrome coronavirus 2* (SARS-CoV-2) and *Clostridioides difficile* (CD) co-infected patients. Recent studies have highlighted the intestinal microbiota’s crucial role in the immune response to SARS-CoV-2 infection by modulating the inflammatory response [1,2]. In this context, fecal microbiota transplant (FMT) may present itself as a beneficial intervention due to its known capacity for reducing circulating inflammatory markers as well as complications and recurrence rates in CD infections (CDI). These effects might be even more notable in CDI patients co-infected with SARS-CoV-2 as both infections are known to elicit exaggerated inflammatory responses. The spike (S) glycoprotein of SARS-CoV-2 binds to its receptor, angiotensin-converting enzyme 2 (ACE 2), and transmembrane protease serine 2 (TMPRSS2), which, at high levels, activates the viral S protein, cleaves ACE2 receptors, and facilitates viral binding to the host cell membrane, causing dysbiosis. In addition, SARS-CoV-2 can also utilize the phagocytosis or endocytosis functions of host cells to invade certain immune cell types, such as macrophages, thus mediating the coronavirus’s entrance into the host cell through membrane fusion. ACE 2 is found in human cells in the intestines, lungs, heart, and kidneys [3]. Endoscopy studies have revealed the presence of colon damage in these patients. Moreover, a biopsy performed on a COVID-19 patient found evidence of the SARS-CoV-2 protein coat in the stomach, duodenum, and rectum. Consequently, host microbiome interface impairment and dysbiosis, along with the inflammatory response, enhance the risk of pathogenic invasion, which might explain the susceptibility of COVID-19 patients to CDI colitis or other pathogens’ co-infection [3,4,5,6]. Moreover, recent metagenomic analyses in COVID-19 patients revealed a marked dysbiosis [7,8,9,10]. A positive correlation has been found between the disease’s severity (along with its pulmonary complications) and the overgrowth of pathogenic bacteria (*Coprobacillus*, *Clostridium ramnosum*, and *Clostridium hathewayi*) to the detriment of *Faecalibacterium prausnitzii* bacteria growth [7]. The latter has shown beneficial anti-inflammatory effects in its hosts’ microbiome population. Furthermore, previous studies suggest that FMT, probiotics, and prebiotics may have a positive role in the treatment of CDI by reducing the incidence of fatal outcomes [7,8,9,10,11]. 

Several reports have highlighted the fact that SARS-CoV-2 patients who are also co-infected with CD complicate clinical management by altering the onset and the clinical course of CDI [1,2,10,12]. In this context, FMT might have a crucial role in the treatment of CDI to improve the long-term evolution and relapse risk [10,11,12,13,14,15,16,17,18,19,20,21]. There is a need for new and innovative treatment approaches to alleviate the inflammatory response. In this sense, further research approaches should be validated by a decrease in inflammatory markers such as C-reactive protein (CRP), white blood cell (WBC) count, procalcitonin, fibrinogen, and d-dimers. Current literature is scarce in this respect. Albeit a few small sample cohort studies have reported FMT outcomes in CDI patients; very few patients with COVID-19 co-infection were enrolled in these studies (case reports on two patients). These studies concluded that FMT appears to be safe and of comparable efficacy in treating recurrent CDI in patients with coexisting COVID-19 and that FMT mitigated more adverse outcomes of SARS-CoV-2 [12,15,22,23,24,25,26].

Moreover, other reports focus mainly on the clinical perspective, providing recommendations for COVID-19 testing (donors and patients) and emphasizing the experience with FMT for recurrent *Clostridioides difficile* infection (rCDI) [27,28] or as a first-line therapy in primary CDI [11,13]. On the other hand, there are several important reports tackling the inflammatory response in SARS-CoV-2 and CDI-infected patients, albeit solely from the perspective of antibiotic therapy and lacking data on the FMT approach [1,2].

The main objective of our study was to highlight the beneficial effects of FMT in patients with SARS-CoV-2 and CD co-infections. We aimed to point how importance it is to repopulate the gut microbiome in order to restore immune function, decrease systemic inflammation, and reduce the risks of recurrence. In addition, we also wanted to analyze and report the safety and clinical benefits of FMT as a first-line therapy in patients with co-infections of SARS-CoV-2 and CDI in comparison with a control group treated only with antibiotics. 

## 2. Materials and Methods

### 2.1. Study Design

Our retrospective, single-center study included 86 patients (46 in the study group vs. 40 in the control group) admitted between January 2020 and March 2022 in the Gastroenterology Department of the County Community Emergency Hospital of Sibiu. This study was performed in accordance with the Helsinki Declaration of 1964. Both groups consisted of COVID-19 and CD co-infected patients. The study was approved by the Ethics Committee in Scientific Research of the Lucian Blaga University of Sibiu, approval code nr.15, approval date 14 October 2022.

The study group included patients receiving FMT and antibiotics at the first episode, while the control group was comprised of patients treated only with antibiotics. We selected the following inclusion criteria, similar to [1,2,11,13,29]: 1. patients over 18 years old; 2. COVID-19 patients (diagnosed by SARS-CoV-2 nucleic acid detection by real-time reverse transcriptase polymerase chain reaction from nasopharyngeal and oropharyngeal swabs); 3. microbiological evidence of CDI (A/B positive toxins); 4. patients who have followed the standard treatment protocol in cases of co-infection: a 10-day course of antibiotics with vancomycin 250 mg × 4/day. After 10 days, if needed, metronidazole 500 mg × 3/day i.v. was added along with vancomycin to control the symptoms. This approach has been implemented with good results by Rokas et al., which showed improved mortality in critically ill patients with CDI when i.v. metronidazole was added to oral vancomycin [30]. The control group received only antibiotic therapy, while in the study group, one FMT infusion was employed after the first 10 days of treatment. These patients ceased antibiotic treatment 24 h prior to the instillation procedure. The decision to administer FMT during the first episode of CDI was made in a similar manner to [11,12] and considered the presence of pseudomembranous colitis at endoscopy as an indication in this regard.

### 2.2. Patient Demographics and Clinical Characteristics

Data collection was procured from the electronic medical records available upon request according to the institutional procedures for clinical retrospective studies. Demographic characteristics included age and gender. We selected the records of patients who had signed informed consent to participate in research studies, similar to [11]. Our focus was to retrieve clinical data related to risk factors, symptomatology, and inflammatory biomarkers. We looked for: (1) comorbidities (malignant neoplasms, diabetes mellitus, cardiovascular diseases, chronic digestive diseases, renal insufficiency, and stroke), (2) clinical manifestations and paraclinical features (number of stools initially assessed and post-transfer of fecal microbiota; abdominal pain which was scaled (mild pain, moderate pain, severe pain), the presence of pseudomembranous colitis at endoscopy—similar or in accordance to data documented in [11,12,21,24,25,26,31,32,33], and biological markers for inflammatory syndromes (WBC count, CRP, and fibrinogen).

The clinical form of COVID-19 infection for the patients included in the study was defined according to World Health Organization (WHO) guidelines as either moderate or severe. There were no patients with mild symptoms admitted, and those with critical disease due to COVID-19 were transferred to the ICU. Moderate pneumonia was defined as the presence of fever, cough, dyspnea, and SpO2 of ≥90% on room air. The severe form of pneumonia was defined by the presence of clinical signs of pneumonia and one or more of the following: a respiratory rate of >30 breaths/min, severe respiratory distress, or an SpO2 of <90% on room air, and increased inflammatory markers (CRP, ESR) [1,2,34].

### 2.3. Fecal Microbiota Transplantation Procedure

First- or second-degree relatives were selected as potential donors according to the current guidelines [35,36]. The fecal matter samples were tested coproparasitologically and virologically (Ag HBS, ACVHC, HIV, CMV, and CD), as well as for COVID-19. Donors were tested for SARS-CoV-2 (by RT-PCR test) [11,37] and all subjects who tested positive were excluded. In addition, patients with documented autoimmune or other chronic diseases and patients who had undergone major surgery or received blood products 12 months before fecal matter collection were also excluded. Donors over the age of 50 were also excluded. We thoroughly analyzed the eligibility of each donor based on their medical records and a personal interview [35,36].

The day before FMT, the patients were prepared by intestinal lavage with 4 L of PEG (Poly Ethylene Glycol). 

**Figure 1:**
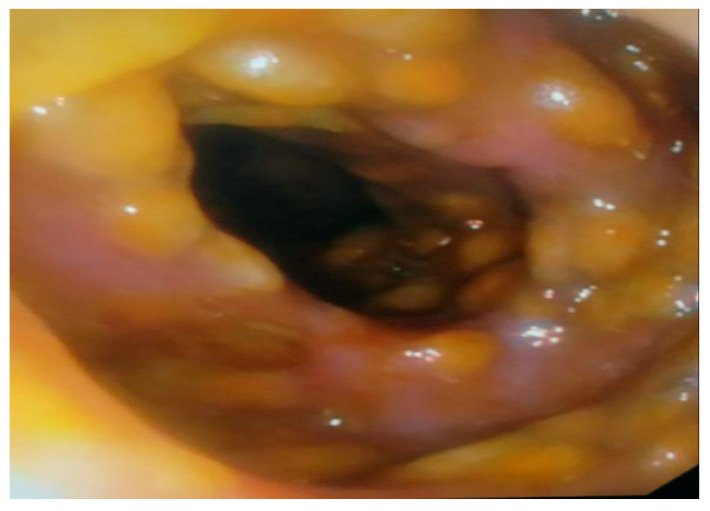
Endoscopic picture before FMT, personal collection.

**Figure 2:**
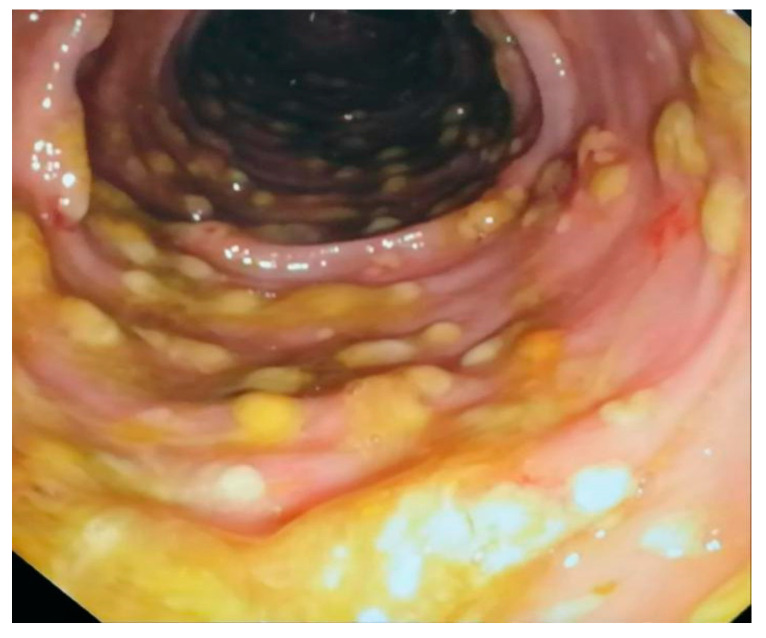
Endoscopic picture before FMT, personal collection.

Fecal matter transfer technique involved dissolving 50 g of donated feces (less than 6 h after defecation) in 500 mL of saline 0.9%, mixing to obtain a homogeneous solution, and filtering. Immediately after, a total colonoscopy was performed with terminal ileoscopy, without sedation, in order to preserve anal sphincter control. The suspension, in its fresh state, was introduced from the terminal ileum, with 2/3 of the suspension in the right colon and the rest in the other segments of the colon at withdrawal. Endoscopic appearance of the colon before fecal microbiota transplant was performed, a typical endoscopic appearance of pseudomembranous colitis was outlined at colonoscopy at the moment of the FMT, despite antibiotic treatment. The colonic mucosa presented erased luster, erased vascularization, granular mucosa, and yellow-green pseudomembranous deposits. Classification of pseudomembranous lesions can be made based on the degree and depth of inflammatory changes, with grading of lesions from type 1 (“summit lesions”, focal surface epithelial inflammation or necrosis) (Figure 1 and Figure 2) to type 3 (mucosal necrosis and significant inflammatory debris) [38].

Safety precautions for endoscopy staff during the COVID-19 pandemic were implemented according to current guidelines established by the World Health Organization and international gastroenterology organizations [34,39,40,41,42]. These precautions included practicing strict physical distancing and minimizing the number of staff in the procedure room. Additionally, all of the staff performing colonoscopies on COVID-19 and CDI patients wore full personal protective equipment, including a respirator (FFP2, FFP3, N95, N99, N100, or equivalent), gown, goggles, face shield, gloves, and apron, while also applying standard precautions in providing patient care.

### 2.4. Outcomes

There were three primary outcomes for our study. The first was based on the reduction of inflammatory markers. The second was related to the resolution of abdominal pain after treatment. Finally, the third outcome concerned disease recurrence, defined in accordance with the European Society of Clinical Microbiology and Infectious Diseases: 2021 update on the treatment guidance document for *Clostridioides difficile* infection in adults [43] as appearing within 8 weeks after a previous episode, after completion of initial treatment [34,43,44]. In consequence, we established an 8-week follow-up period in our study after the patients’ first episode of CDI and subsequent treatment.

The secondary outcome was related to adverse effects attributed to the FMT procedure.

### 2.5. Data Analysis 

We performed univariate analyses of continuous and categorical variables. Continuous variables were represented by age (in years) and inflammatory biomarkers (white blood cell (WBC) count, CRP, and fibrinogen). Categorical variables referred to gender, comorbidity categories (diabetes mellitus, neurological diseases, cardiovascular diseases, obesity), clinical manifestations, and paraclinical features (pseudomembranous colitis at endoscopy, number of stools, abdominal pain). Categorical variable analyses and results were presented as frequencies and percentages, while continuous variables were presented as means and standard deviations. Groups were compared using the chi-square or Fischer test for categorical variables and the independent *t*-test or Mann-Whitney test for continuous variables. An α-level of 0.05 was considered statistically significant.

## 3. Results

### 3.1. Study Population and CDI Risk Factors

There was a total of 235 patients with SARS-CoV-2 and CD co-infection documented during the two-year period of our retrospective search. 149 cases were excluded due to one or more of the following: missing informed consent to participate in research studies; missing data (particularly on recurrence rate); death during hospitalization or transfer to ICU; significant comorbidities or demographic characteristics which would have created inhomogeneities between groups or which would have had an intrinsic influence on the determined outcome variables.

We recorded a notable difference in patients coming from rural areas (60.5%) vs. the patients from urban environments (39.5%), however, with the same distribution pattern in both groups (*p* > 0.05). On the same note, both groups were homogeneous, with the same percentage of patients (60.4%), hypertension as a factor of severity, and diabetes mellitus type 2. Then, in both groups, there was a close distribution of neurological and metabolic risk factors. Around 7% of the cases presented a stroke in their medical history, and obesity as a risk factor was noted in a slightly higher percentage for the study group (17.4% vs. 15%) (*p* > 0.05) (Table 1).

### 3.2. Clinical Manifestations and Inflammatory Status Prior to Treatment

There were no significant differences between patients with regards to their symptoms (i.e., moderate vs severe abdominal pain and number of stools per day) or the inflammatory status (quantified by WBC count and CRP) prior to treatment, as shown in Table 2.

With regards to COVID-19 disease severity, there were 20 patients (50%) in the control group with moderate disease and 20 patients with severe disease. In the FMT group, 23 patients presented with a moderate form (50%), and 23 had a severe form.

### 3.3. FMT’s Role in Decreasing the Inflammatory Syndrome, Abdominal Pain, and Risk of Recurrence

We recorded a statistically significant improvement in inflammatory syndrome (CRP, WBC count) after FMT, *p* < 0.05. In the control group, 29 patients (72.5%) received Metronidazole and Vancomycin, in comparison with the study group, where this combination of antibiotics was used only in 1 patient (2.17%). There was only one case of relapse (2.17%) out of the 46 patients receiving an FMT, in contrast to 17 cases of recurrence (42.5% relapse rate) in the control group (*p* < 0.05).

Post-treatment more than 91% of FMT patients had no abdominal pain while a significant number of patients treated only with antibiotics (82.5%) had persistent moderate pain (*p* < 0.05). (Table 3). FMT alleviated abdominal pain and lowered the relapse rate (*p* < 0.05). 

**Figure 3:**
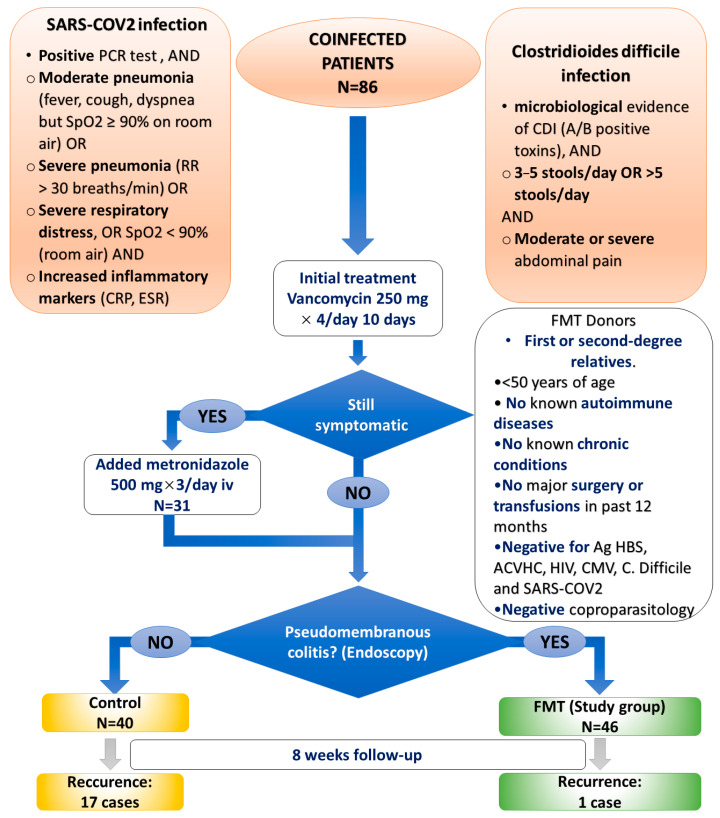
Study design and results regarding disease recurrence.

The study design and a representation of the results concerning disease recurrence are given in Figure 3.

With regards to our secondary outcome, we did not record any severe adverse effects during or after the FMT procedure for the 46 patients that underwent this intervention.

## 4. Discussion

In this paper, we analyzed the importance of FMT in alleviating gut microbiome dysbiosis and improving immune system function in SARS-CoV-2 and *C. difficile* co-infected patients. We hypothesized a decrease in systemic inflammation along with improvements in clinical condition and a reduction in the recurrence risk for these patients. To achieve this goal, we analyzed and reported the safety of FMT and its clinical benefits as a first-line therapy for the aforementioned patients, in comparison to the control group treated only with antibiotics. 

Our results showed that in more than 90% of the whole cohort (study and control groups), specific risk factors such as stroke, diabetes, obesity, and hypertension were present as important comorbidities for both infections, which is consistent with the current literature [11,12,16,17,19]. Our results are in line with Kovacevic et. al. [45] who recorded similar risk factors for co-infection (cardiovascular disease (11.96%) and diabetes (30.37%)).

*Clostridioides difficile* preferentially affects elderly patients and causes a high mortality rate, especially in co-infection. In the literature, the co-infected patients’ profile has a mean of 61.22 years of age, comes from urban areas, and has a higher chance of being female (62.5%). In contrast, our study noted homogeneity in gender prevalence in the included population. In addition, a higher percentage of patients were from rural areas, albeit without reaching statistical significance (*p* > 0.05) [1,2,16,22]. Rajib et al. outlined smoking rates and obesity rates explain, to some extent, the geographic disparities in COVID-19 prevalence. Their study noted a higher obesity rate in rural areas and a higher percentage of adult smokers, increasing the risk for cardiorespiratory diseases, but further studies are needed in terms of epidemiology [46]. However, our study did not assess smoking status and showed a homogenous distribution of the studied comorbidities.

Recent clinical trials mention the role of FMT in inflammatory bowel disease (IBD), multiple sclerosis, and Parkinson’s disease. This suggests not only a local modulating intestinal effect but also a systemic immunological response to FMT with an impact on the gut-lung and gut-brain axes [3,46]. The immunological response after FMT was associated with a substantial reduction in the colonic mucosal CD8+ T cell density and a decrease in serum concentrations of IL-6, and IP-10. Serum levels of IL-6 and VCAM-1 were all significantly correlated with CRP and ESR, as has been highlighted in the study by Yanzhi et al. on FMT’s role in ulcerative colitis treatment [47]. Given the excessive inflammatory response in SARS-CoV2 and CDI infections, with elevated levels of IL-6, TNF-α, IL-1β, and IP-10, as well as associated dysbiosis mentioned in the literature [3,4,48], FMT might be a key factor in CDI treatment or more severe clinical scenarios like SARS-CoV-2 and CDI co-infections. FMT could improve the clinical outcome of these patients.

Clinical evolution for CDI and/or SARS-CoV-2 patients is related to the associated inflammatory response. According to Clinical Practice Guidelines for *Clostridioides difficile* Infection in Adults and Children: 2017 Update by the Infectious Diseases Society of America (IDSA) and Society for Healthcare Epidemiology of America (SHEA), an important cut-off point is leukocytosis (>15.000 cells/mL). Our baseline mean in leukocytosis (16.613 cells/mL) could be regarded as a crucial predictor for the clinical outcome and survival [16,19,22,23]. Moreover, Konturek et. al. outlined a significant CRP reduction after FMT therapy in all treated patients [20]. Likewise, our data showed normalization of inflammatory markers: CRP 5.67 mg/dl mean, WBC mean 7695 cells/mL, and fibrinogen 420 mg/dl in FMT patients (*p* < 0.05). 

It is acknowledged that SARS-CoV-2 and CDI co-infection challenge clinical management in the sense that abdominal cramping and pain may be more severe and longer lasting after the disease’s resolution [20,33,38]. We recorded a statistically significant correlation between FMT and the reduction of abdominal pain for our patients at discharge (91.3%, *p* < 0.005). In a similar approach, other studies focusing on CDI alone pointed out a significant reduction in abdominal pain and bloating at 4–8 weeks. Furthermore, a retrospective clinical review regarding FMT for CDI infection also highlighted a significant improvement in abdominal pain and cramping from baseline at 6-month follow-up [17,22,45]. These aspects, in addition to the data presented in our study, suggest that restoring a normal microbiota through fecal instillation relents abdominal pain and improves patients’ quality of life. 

On the same note, the FMT group needed only one class of antibiotic (vancomycin) in most of the cases (97.82%) with a better clinical evolution, while the control group, in 75% of the cases, received a second antibiotic class (metronidazole) associated with properly managing the case. Similarly, previous studies on fecal microbiota transplantation using colonoscopy pointed out a significantly more effective approach than antibiotherapy (vancomycin) per se or vancomycin and fidaxomicin to abate the clinical symptoms of CDI [18]. The literature is scarce, however, when it comes to SARS-CoV-2 patients and CDI. To the best of our knowledge, there are only a few reports that address SARS-CoV-2 and CDI co-infections (case series). Further clinical trials assessing a personalized therapeutic management for these cases could shed light in regards to survival rates, antibiotic use reduction, antibiotic associations, and their side effects. Consequently, our study adds important information to the literature concerning the treatment methodology of SARS-CoV-2 and CDI.

Moreover, many research papers highlighted that CDI was associated with a high risk of relapsing, especially in patients with comorbidities and/or co-infections. In the latest reports, early fecal transplantation was associated with a significantly reduced mortality rate for recurrent CDI. Furthermore, in a Cox model proposed by Lagier et al., early transplantation was the only independent predictor of survival in severe CDI (hazard ratio 0.18, *p* = 0.006) [15].

Our previous results showed important FMT benefits from the first episode in cases of severe colitis, which correlated with the risk of relapsing [11]. In this study, we recorded only one case of CDI recurrence after 6 weeks in the FMT group. Our patient presented with severe leukocytosis, more than six stools/day, and associated diabetes and obesity. In contrast, there were multiple cases of recurrence in the control group—17 patients (42.5%). Similar results were presented by Marinescu et al. outlining the recurrence in 19 co-infected patients treated only with antibiotics [2,18]. Roshan et al. in their retrospective study mentioned relapse in one case 5 weeks after the FMT procedure [17,20,22]. These reports further add importance in providing arguments for FMT being subsequently employed after monotherapy to prevent CDI recurrence [18].

An important overall observation that should be highlighted is the 97.82% success rate that we recorded, regardless of age and comorbidities. In addition, very few adverse effects are generally directly attributable to the procedure. Most reported adverse events in the literature have been self-limiting gastrointestinal symptoms, comprising abdominal cramps, nausea, and constipation. Fever, gram-negative bacteremia, and bowel perforation are very rare adverse effects [19,49,50]. The patients enrolled in our study were informed about these possible adverse effects. We did not, however, record any severe adverse effects during or after the procedure for the 46 patients that received FMT. Recent studies discuss a new method to further improve the safety of FMT, called washed microbiota preparation. This approach is based on the use of an automatic microfiltration machine and subsequent repeated centrifugation. In a study with patients who underwent either washed microbiota transplantation (WMT) or crude FMT in the same FMT center with the same indications, fewer adverse effects were recorded in the WMT group. However, further studies are needed to understand the mechanisms behind these findings [51,52,53]. Consequently, FMT might be regarded as an alternative therapeutic solution, especially in older patients with comorbidities, modulating the immune system’s functionality and increasing the survival rate. 

Nevertheless, these results must be interpreted with caution, and several limitations should be kept in mind, as this is a single-center research report and a retrospective study with the possibility of residual confounding based on the data gathered during the COVID-19 pandemic. Further research using multicentric randomized control trials is needed to establish a treatment protocol for practitioners in co-infection cases.

## 5. Conclusions

The fecal microbiota transplantation approach shows promise regarding the safety and efficiency of the treatment of CDI in patients with co-existing COVID-19. It offers a personalized therapeutic management strategy with multiple benefits like decreasing inflammatory syndrome, limiting the use of antibiotics, reducing relapse risk, and alleviating symptomatology. 

## Figures and Tables

**Table 1:** Demographic and clinical characteristics of the patients.

Variable	FMT Group (*n* = 46)	Control Group (*n* = 40)	Chi-Square Test *p*-Value
Age (years)	67.63	67.68	0.917
Male gender Female gender	23 (50%) 23 (50%)	20 (50%) 20 (50%)	0.585 0.585
Rural areas Urban	33 (71.73%) 13 (28.26%)	19 (47.5%) 21 (52.5%)	0.28 0.28
Cardiovascular disease	6 (13%)	7 (17.5%)	0.817
Hypertension	14 (30.4%)	12 (30%)	0.817
Neurological disease	3 (6.5%)	3 (7.5%)	0.817
Diabetes	14 (30.4%)	12 (30%)	0.817
Obesity	8 (17.4%)	6 (15%)	0.815
Antibiotics			
Vancomycin	45 (97.82%)	11 (27.5%)	0.001
Vancomycin and metronidazole	1 (2.17%)	29 (72.5%)	0.001

**Table 2:** Clinical manifestations and inflammatory status prior to treatment.

Variable	FMT Group (*n* = 46)	Control Group (*n* = 40)	*t*-Test Chi-Square Test
CRP pre-treatment (mean)	46.8 mg/L	56.2 mg/L	0.18
WBC count pre-treatment (mean)	17,000 cells/mL	16,850 cells/mL	0.795
Moderate abdominal pain pre-treatment	26 (56.5%)	18 (45%)	0.28
Severe abdominal pain pre-treatment	20 (43.5%)	22 (55%)	0.28
3–5 stools/day pre-treatment	28 (60.9%)	24 (60%)	0.93
>5 stools/day pre-treatment	18 (39.1%)	16 (40%)	0.93

**Table 3:** Relapse rate, inflammatory markers, and abdominal pain after treatment between study groups.

Variable	FMT Group (*n* = 46)	Control Group (*n* = 40)	*T* Test Chi-Square Test * Fisher Test
Fibrinogen	420 mg/dl	452.55 mg/dl	0.02
CRP post-treatment (mean)	5.67 mg/L	9.18 mg/l	0.001
Leucocyte post-treatment (mean)	7695 cells/ml	85,751 cells/mL	0.001
No abdominal pain post-treatment	42 (91.3%)	7 (17.5%)	0.0001
Moderate abdominal pain post-treatment	4 (8.7%)	33 (82.5%)	0.001
Relapse	1 (2.2%)	17 (42.5%)	0.001 *

## Data Availability

The data presented in this study are available on request from the corresponding author.
